# Cold stimuli, hot topic: An updated review on the biological activity of menthol in relation to inflammation

**DOI:** 10.3389/fimmu.2022.1023746

**Published:** 2022-11-09

**Authors:** Haojin Cheng, Xuemei An

**Affiliations:** ^1^ College of Clinical Medicine, Chengdu University of Traditional Chinese Medicine, Chengdu, Sichuan, China; ^2^ Nursing Department, Hospital of Chengdu University of Traditional Chinese Medicine, Chengdu, Sichuan, China

**Keywords:** menthol, inflammation, review, TRPM-8, natural products

## Abstract

**Background:**

Rising incidence of inflammation-related diseases is an increasing concern nowadays. However, while menthol is a wildly-used and efficacious complementary medicine, its pharmacological mechanism still remains uncertain. Superimposed upon that, the aim of this review is to summarize the contemporary evidence of menthol’s anti-inflammatory activity.

**Methods:**

Using the pharmacopeias and electronic databases, including Web of Science, PubMed, and CNKI, this study analyzed the relevant research articles and review articles from 2002 to 2022 and concluded those results and conjectures to finish this article.

**Results:**

The decrease in pro-inflammatory cytokines and related inflammatory markers, as well as associated pathway activation, was found to play the greatest role in the protective effects of menthol against inflammatory damage or association with protection against chronic inflammation.

**Conclusion:**

This review mainly concludes the progress in menthol’s anti-inflammatory activity. Further studies are needed to establish relationships between the mechanisms of action and to clarify the clinical relevance of any anti-inflammatory effects.

## Introduction

Menthol is a monocyclic monoterpene that is obtained from plants of the genus Mentha as an essential oil (1). Menthol was first isolated in 1771 by the German, Hieronymus David Gaubius. Early characterizations were done by Oppenheim, Beckett, Moriya, and Atkinson. It was named by F. L. Alphons Oppenheim (1833–1877) in 1861. In general terms, when menthol is mentioned, it usually refers to L-menthol. Menthol has become ubiquitous in a range of products due to the minty taste and scent of the compound and its cooling or anesthetic effect when applied to the skin or mucosal membranes (1). Consequently, menthol is found in tea, cosmetics, foods, pharmaceutical products, healthcare products, and tobacco products (2). While widely recognized in the form of essential peppermint oil, the synthesis of menthol on an industrial scale is diverse, reflecting its use in a variety of products (3). Increasingly, there is an interest in determining how menthol use may be diversified in healthcare contexts in particular, given the evidence for cooling and anesthetic effects, as well as an increasing recognition of wider effects in the body (4). While it has been noted that menthol influences the vascular system, immune system, and neurological system for decades, there remains uncertainty over the specific activities of menthol from a therapeutic perspective (5).

**Figure 1:**
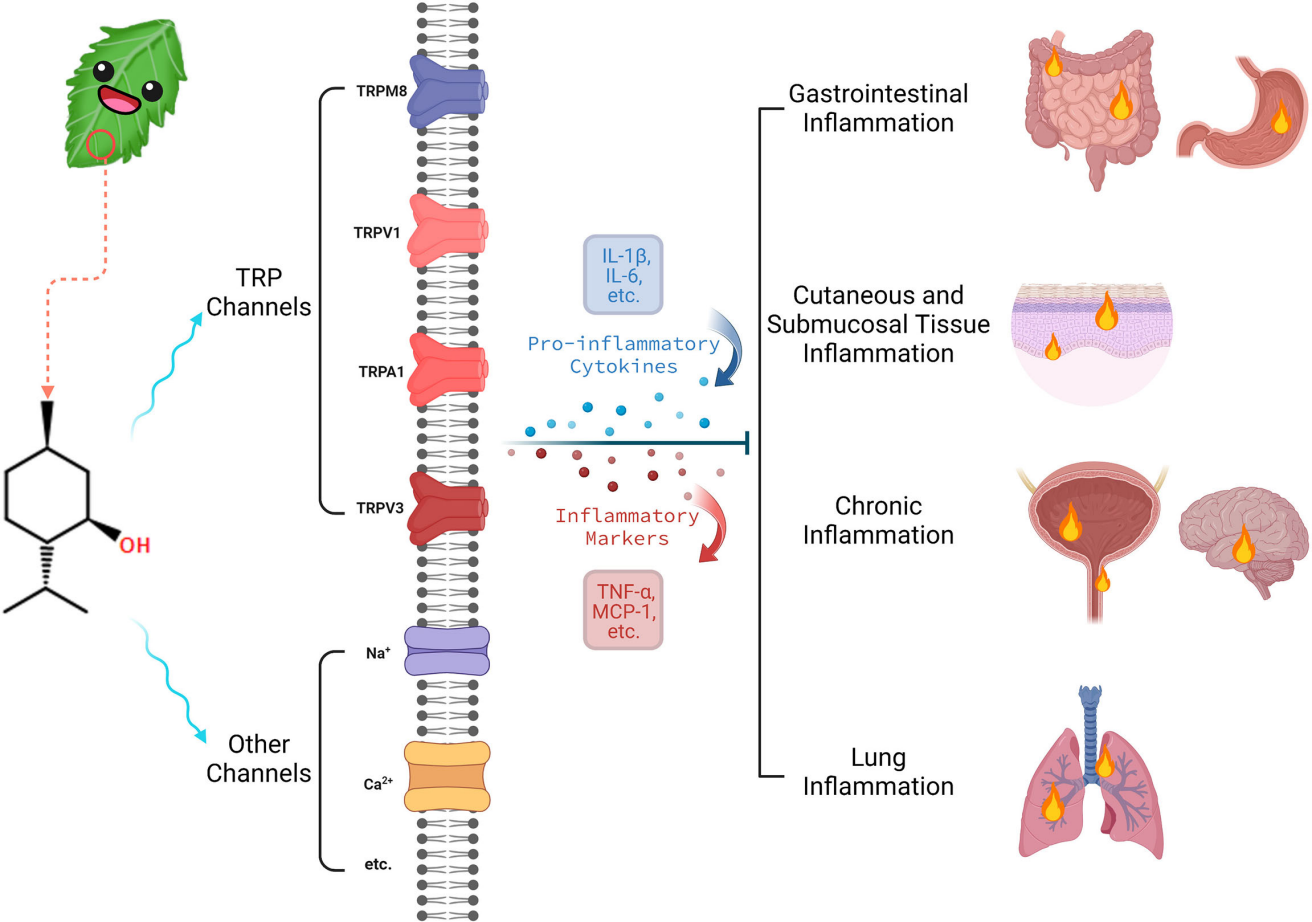
Brief overview of menthol’s biological activity in inflammation.

One emerging area of interest where menthol may be applied therapeutically is in the context of inflammation (6). Inflammation is a complex process that is the culmination of a cascade of immune responses that promote changes to local tissue (7). Inflammation may be a biologically advantageous reaction to an infectious agent or other pathological processes, increasing blood flow to the affected region (8). However, chronic inflammatory states are increasingly recognized as contributing to pathological conditions, including links to cardiovascular disease, obesity, and diabetes (9). Acute inflammatory responses may also be associated with discomfort, pain, or functional impairment (10). The diverse effects of menthol and the well-established influence of the compound on cooling sensations when applied to the skin, notably in inflammatory conditions, suggest that the role of menthol in inflammation requires further investigation (6). The remainder of this paper provides a detailed review of the literature in relation to the biological activity of menthol, with a focus on the role of menthol in inflammatory pathways. **Figure 1**, created with Biorender.com, provides a brief overview of this paper.

## Chemical and physical properties of menthol

**Figure 2:**
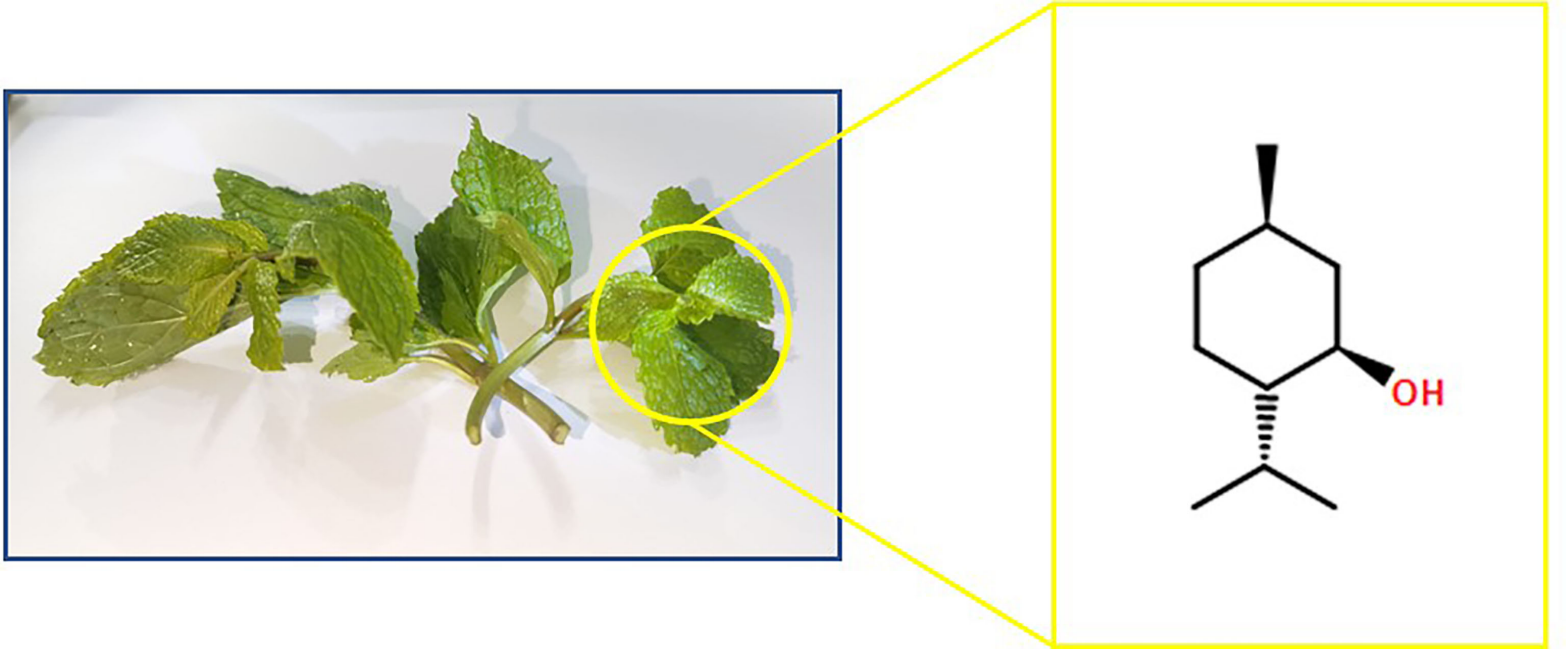
Chemical formula of L-menthol from mint source.

Menthol, with the chemical formula C_10_H_20_O and a molar mass of 156.27, is a naturally occurring compound with three asymmetric carbon atoms. Thus, menthol often occurs as four optical isomers: menthol (L-menthol), neomenthol, isomenthol, and neoisomenthol. The chemical formula of L-menthol is illustrated in **Figure 2**. Menthol occurs in nature in the vast majority of cases as L-menthol. Similar to other saturated alcohols, menthol is capable of reacting and being oxidized to menthone in a variety of forms. Menthol is a white or colorless solid or crystalline flake at room temperature with a density of 0.890 kg/dm^3^. Menthol is completely insoluble in water but freely soluble in ethanol, diethyl ether, or chloroform. Similar to citronellol and geraniol which can be extracted from geranium oil, rose oil, and many other essential oils, menthol does not absorb UV light at 290–32-pinene 0 nm, but begins to absorb below 290 nm and peaks at 220 nm (3).

As a naturally occurring cyclic monoterpene, menthol may utilize its chemical structure (e.g., hydroxyl groups) for its potent antioxidant effects. Several previous studies have concluded that the major monoterpenoids, particularly menthol, are responsible for the majority of the mint’s antioxidant activity (11, 12). In general, phytochemicals exert their antioxidant effects by scavenging free radicals, chelating divalent metals, donating hydrogen or electrons, and facilitating the decomposition of peroxyl radicals. As a result, phytochemicals can inhibit the formation of free radicals, slow or inhibit the autoxidation process (chain-breaking antioxidant), or accelerate the termination of autoxidation. Wu et al. (12) use *in vitro* chemical- and cell-based assays and *in vivo* tests with *C. elegans* model to prove that the major monoterpenoids of mint essential oil, particularly menthol, have potent antioxidant effects.

## Biological activity of menthol: receptor activity and signaling pathways

As a simple monoterpene with remarkable biological properties, menthol possesses well-known cooling properties and a residual minty scent from the oil remnants. It is also known to exhibit biological activity *in vitro* and *in vivo*, including anti-inflammatory, antibacterial, analgesic, antipruritic, anticancer, and antifungal effects.

Menthol has been identified as an agonist of transient receptor potential melastatin-8 (TRPM8) receptors in the epithelium (13). These receptors, also termed cold receptors, are linked to the sensation of a cooling effect on skin and mucosal membranes when activated (14). TRPM8 is considered a key receptor in eliciting the sensation of “cold” in human skin (15). The effects of menthol *via* the TRPM8 receptor include an analgesic or anesthetic effect related to the cooling process. While the mechanism of action of menthol in this context is not completely understood, it is thought that downstream TRPM8 signaling disrupts typical nociceptive nerve signaling and associated pathways, accounting for pain alleviation following menthol oil application (16). Knockout of the TRPM8 receptor in mice leads to the lack of perception of cold environmental sensations and the lack of menthol-induced analgesia when assessed in the context of acute or inflammatory pain (13). Structural analysis of the TRPM8 receptor suggests that the defined domains and large cytosolic ring have extensive interactions with the transmembrane channel, suggesting the ability to transduce significant chemical or environmental stimuli into downstream signaling events (16).

Menthol also acts through other channels, independently of activity linked to TRPM8 agonism, suggesting the potential for diverse biological effects. Menthol can block voltage-gated sodium channels, which play important roles in the production and propagation of action potentials in excitable cells in a concentration-, frequency-, and voltage-dependent way (17, 18). Several relevant reports have demonstrated that menthol reduces Ca^2+^ inflow through the low voltage-activated Ca^2+^ channel while increasing the inactivation of the high voltage-activated Ca^2+^ channel (19–21). In addition, Umezu (21) also noticed that L-menthol inhibited the binding of (-)-desmethoxyverapamil to Ca^2+^ Channel at the rate of 24.78%, but rather low inhibition rate to the radioactive isotope-labeled ligands, including PN200-110, (+)-cis-Diltiazem, and ω-Conotoxin GVIA. There’s also evidence showing that menthol appears to promote Ca^2+^-activated K^+^ currents in human glioma cells, but the relationship between menthol and the K^+^ channel still remains unclear due to insufficient research data (22). Vogt-Eisele et al. (23) found that menthol can activate the transient receptor potential vanilloid 3 (TRPV3) in the skin and neural tissues, which is linked to the sensitization of the skin. Analgesic effects of menthol have also been ascribed to the activation of transient receptor potential vanilloid 1 (TRPV1) and transient receptor potential ankyrin 1 (TRPA1) channels, often overlapping with the activation of TRPM8 receptors in the skin (24). Menthol has also been reported to interact with nicotinic acetylcholine receptors in the respiratory tract, non-competitively antagonizing their activity (25). While this effect is not completely understood and has not been evaluated in detail, it is proposed that the soothing effects of menthol on the airways during cigarette smoking may be due to blocking the nicotine-receptor interactions that have been associated with pain and inflammation in this respiratory tract (25).

The biological effects of menthol have generally been linked to analgesic effects when topically applied to the skin, as well as an increasing interest in respiratory or systemic effects following inhalation due to the popularity of menthol cigarettes (26). Analgesic effects *via* the TRPM8 receptor and associated TRP channels, are well described and are closely related to the cooling effects seen upon TRPM8 activation (6). Nevertheless, the role of menthol in inflammatory pain relief and in alleviating inflammatory responses associated with cigarette smoking has raised interest in the potential anti-inflammatory mechanisms of this compound (14). The remainder of this paper considers the influence of menthol on inflammation in available studies to appreciate the effects of menthol and the mechanisms accounting for those effects.

## Influence of menthol on inflammation

### Overview

Numerous animal studies and experimental designs have explored the anti-inflammatory effects of menthol in a series of disease contexts. A literature search was completed using online journal databases to identify primary studies exploring the activity of menthol on inflammatory pathways and conditions. This section provides an insight into the literature, with a focus on the influence of menthol on markers of inflammation and inflammatory pathways, grouped according to tissue type or anatomical location to allow for comparability of studies.

### Anti-inflammatory activity of menthol in models

By summarizing the results of the current study in several online journal databases, we have organized the brief information of human, *in vivo*, and *in vitro* studies showing the biological effects of menthol on inflammation in **Table 1**.

**Table 1:** Anti-inflammatory activity of menthol examined by human, *in vivo*, and *in vitro* studies.

Main outcomes	Species/Subjects	Menthol dose, route, and duration of administration	specific organ/system	References
↑TNF-α, IL-1β, and p-mTOR expression ↑intracellular Ca2+ concentration	human bronchial epithelial cells (BEAS-2B)	1 mmol/L, culture medium, 0.5, 1, 3, 6, 12, 24 hours	bronchus	Chen and Li (27)
↓the release of TNF-α	THP-1 cell line	1% ethanol concentration, culture medium, 2 hours	THP-1 cell	Hilfiger et al. (28)
↓TNF-α expression ↓phosphorylation of p38 ↑IL-10 ↑phosphorylation of the extracellular-signal-regulated kinase ↑uptake of zymosan particles ↓cells with high phagocytic activity (more than three bound/ingested particles) ↓cell migration	*in vitro*: F4/80+ macrophages *in vivo*: F4/80+ macrophages from WT and TRPM8-mutant C57BL/6 mice	*in vitro*: 100 μmol/L, culture medium, 1h *in vitro*: 100 μmol/L, culture medium, 8h *in vivo*: 100 μmol/L, enemas, twice a day for 7 days	colon	Khalil et al. (29)
↓acrolein-induced TRPA1-dependent injury behavior ↓mechanical hyperalgesia	wild-type mice	50 nmol/50 μl, intraplantar injection, 10 minutes. 60 nmol/20 μl, intraplantar injection, 30 minutes before testing	plantar surface of the hind paw	Liu et al. (6)
↓sensory irritant response to acrolein ↓cyclohexanone-induced irritant response ↓respiratory irritation response ↑blood cotinine levels ↓active phases of inspiration and expiration	female C57Bl/6J mice	Inspired concentration of 8, 40, 54 ppm, inhalation, 15 minutes	lung	Ha et al. (30)
↓expression of COX-2 ↑level of GSH ↑activities of antioxidant enzymes including CAT, GST, GR and GPx ↓expression level of NF-κB ↓expressions of p38 and ERK	48 female ICR mice	20 and 80 mg/kg, applied onto the shaved area of dorsal skin, twice a week for 20 weeks	skin	Liu et al. (31)
↑expression of TRPM8 ↑abnormal re-epithelialization, thickening of alveolar walls, and infiltration of inflammatory cells ↑BALF total protein ↑BALF total cell counts ↑BALF lymphocyte cell and neutrophil cell ↓BALF macrophage cell ↑BALF MIP-2 ↑lung MIP-2 ↑expression of 4-HNE ↑expression of ERK and JNK	male C57BL/6J mice	750 mL of fresh smoke generated from 1.5 Marlboro Black Menthol cigarettes, inhalation, 20 minutes per day for 1 week	lung	Lin et al. (32)
↓intensity of Ca^2+^ influx ↓expression level of TRPV1 ↑body weight and food consumption ↓hyperplasia of the esophageal epithelium ↓levels of the inflammatory factors TNF-α, IL-6, and IL-1b	Male C57BL/6 J mice	50 mg/kg, p.o., 21 days	esophagus	Zhang et al. (33)
↑cumulative survivals ↓cTnI levels ↓LVEDD and LVESD ↑EF and FS ↑dP/dt_max_ and dP/dt_min_ ↓elevation of neutrophil density and macrophage density ↓elevation of TNF-α, IL-1β, IL-6, MCP-1 ↑release of calcitonin gene-related peptide	heterozygous knockout mice (generated by breeding TRPM8^-/-^ mice with C57BL6/J wild-type mice)	0.5%, mixed in chow diet as pre-treatment, 2 weeks	heart	Wang et al. (34)
↑plasma cotinine concentrations ↓total and nonrenal clearance of nicotine ↓The ratio of nicotine glucuronide to nicotine in the urine	14 healthy smokers	61.4 mg/d, inhalation, 1 week	lung	Benowitz et al. (35)
↓course of complete healing in napkin dermatitis ↓Erythema and pustules	70 neonates with a diagnosis of candidial napkin dermatitis	5% ethanol concentration, external skin use, twice a day for 7 days	skin	Sabzghabaee et al. (36)
↓average ulcer area ↓infiltration of neutrophils into the gastric submucosa ↑expression of HSP-70 ↓expression of the apoptotic protein Bax ↓immunolabeled area ↓MPO activity ↑total glutathione levels ↑glutathione peroxidase activity ↑glutathione reductase activity ↓SOD enzyme ↓levels of cytokines ↓levels of TNF-α and IL-6 ↑level of IL-10	21 male Wistar rats	50mg/kg, p.o., 2 hours	stomach	Rozza et al. (37)
↑cold detection threshold and cold pain threshold ↑cold hyperalgesia assessments ↓warmth detection threshold and heat pain threshold ↓thermal sensory limen ↑superficial skin perfusion	10 healthy subjects	40% ethanol concentration, external skin use (volar forearm), 20 minutes	skin (volar forearm)	Olsen et al. (38)
↓area of secondary mechanical hyperalgesia ↓secondary neurogenic flare	14 healthy subjects	40% ethanol concentration, external use, 20 minutes	skin	Andersen et al. (39)
↓itch intensity (except for 32°C and 37°C) ↓neurogenic inflammation ↓wheal reactions	13 healthy subjects	40% ethanol concentration, external skin use, 7 minutes before itch induction	skin (volar forearm)	Andersen et al. (40)
↓loss of body weight ↓macroscopic damage score, ulcer area, colon weight ↓colon length ↑reepithelization of the mucosal layer ↓edema ↓inflammatory cell infiltration in lamina propria ↑hematocrit ↓changes in MPO activity ↓IL-1β, IL-6 and TNF-α	36 male Wistar rats	20, 50, and 80 mg/kg, i.p., once a day for 3 days	colon	Ghasemi-Pirbaluti et al. (41)
↓lung injury (with decreased values of mean linear intercept and destructive index) ↓decrease in values of body weights ↑spleen index ↓levels of AST and ALT ↓numbers of total white blood cells and macrophages ↓TNF-α, IL-1β, IL-6 ↓MPO and MDA levels ↑levels of GSH, GSH-Px, SOD, and T-AOC ↓expression of NF-κB p65 and p-p38 ↑expression of Nrf2 and NQO1 ↓expression of MMP-9, TIMP-1, CD4^+^ and CD8^+^ cells	42 male SPF Sprague-Dawley rats	5, 10, 20 mg/kg, i.p., 3 weeks	lung	Liu et al. (42)
↓IBD-induced reduction in mean body weight, mean macroscopic and microscopic ulcer scores ↓activities of MPO and levels of MDA ↑glutathione level ↓levels of pro-inflammatory cytokines such as IL-1, IL-23, and TNF-α	36 male albino Wistar rats	50 mg/kg, p.o., 7 days and 10 days	colon	Bastaki et al. (43)
↓rotational behavior of LPS-exposed rats ↓decrease in the number of tyrosine hydroxylase-positive cells ↑expression of tyrosine hydroxylase protein ↓microglial activation ↓upregulation of the pro-inflammatory enzymes COX-2 and iNOS at both the gene and protein levels ↓upregulating LPS-induced pro-inflammatory factors such as TNF-α, IL-6, and IL-1β (p38 excluded) ↓phosphorylation levels of NF-κB p65, AKT, ERK1/2, and JNK1/2	Male Wistar rats	40% ethanol concentration, i.p., 4 weeks	microglia	Du et al. (44)
↑mean BBB score ↑paw withdrawal threshold ↓caspase-3 immunoreactivity ↓apoptotic bodies ↑Arg-1 expression ↓GFAP, IBA-1, and TGF-β1 ↓collagen IV ↑brain-derived neurotrophic factor expression ↑expression of the growth cone marker ↓ANGPT-1 expression ↑GAP-43 expression ↓lesion area	female Sprague-Dawley rats	10 mg/kg, i.p., 2 weeks	Spinal Cord	Kim et al. (45)
↓overall mortality ↓MDA and total nitrate/nitrite levels in the lung and kidney homogenates ↓TNF-α expression ↓immunoreactive cells ↑Bcl2-immunoreactivity ↑nuclear proliferating cell nuclear antigen immunoreactivity	28 female Wistar rats	100 mg/kg, p.o., 7 days	lung and kidney	Anter et al. (46)

BBB score, Basso, Baettie, and Bresnahn score; Arg-1, Arginase-1; GFAP, glial fibrillary acidic protein; IBA-1, ionized calcium-binding adapter molecule-1; TGH-β1, transforming growth factor-β1; ANGPT-1, angiopoietin-1; GAP-43, growth-associated protein-43; N2a, Neuro2A; cTnl, cardiac troponin I; LVEDD, left ventricular end-diastolic diameter; LVESD, left ventricular end-systolic diameter; EF, eject fraction; FS, fractional shortening; Dp/dt_max_, maximal rate of pressure development; Dp/dt_min_, minimal rate of pressure decay; TNF-α, tumor necrosis factor-α; IL-1β, interleukin-1β; IL-6, interleukin-6; MCP-1, monocyte chemoattractant protein-1; p-Mtor, phosphorylated-mammalian target of rapamycin; TRPA1, transient receptor potential A1; MDA, malondialdehyde; Bcl2, B-cell lymphoma-2; PCNA, proliferating cell nuclear antigen; MPO, myeloperoxidase; SOD, superoxide dismutase; COX, cyclo-oxygen-ase, Inos, inductible nitric oxide synthase; AKT, protein kinase B; ERK, extracellular regulated protein kinase; JNK, c-Jun N-terminal kinase; GSH, glutathione; GSH-Px, glutathione peroxidase; CAT, catalase; GST, glutathione s-transferase; GR, glutathione reductase; GPx, glutathione peroxidase; BALF, bronchoalveolar lavage fluid; T-AOC, total antioxidant capacity; NF-κB, nuclear factor kappa B; Nrf2, nuclear factor erythroid 2-related factor 2; NQO1, NAD(P)H: quinone oxidoreductase 1.

### Gastrointestinal inflammation

Within the gastrointestinal tract, inflammatory reactions are associated with a number of adverse conditions and pathologies (47). As menthol can be administered orally and is safe for human consumption, the potential for menthol to exert anti-inflammatory effects in the gastrointestinal tract is attractive, provided these effects can be demonstrated in pathological contexts. Evidence to date suggests that menthol may reduce inflammation associated with gastric ulceration (37), gastro-oesophageal reflux-associated inflammation (33), and colitis (41, 43).

Menthol has demonstrated anti-inflammatory effects within the gastric mucosa in an experimental model of ethanol-induced gastric ulcers (37). Ethanol-induced gastric ulcers are characterized by marked inflammatory responses following the generation of reactive oxygen species and a vigorous immune reaction (48). The study found that menthol administration led to an increase in anti-inflammatory interleukin (IL)-10 expression, while there was a reduction in pro-inflammatory markers, including IL-6 and tumor necrosis factor-α (TNF-α). The changes in these inflammatory markers were all statistically significant compared with baseline levels (P<0.001). This study investigated the antioxidant, anti-apoptotic, and anti-inflammatory effects of menthol, providing the potential to differentiate these effects within a model whereby all three factors contribute to ulceration and associated symptoms (37). Importantly, menthol administration led to a reduction in markers of antioxidant activity and a reduction in the neutrophilic immune response in the gastric mucosa, which collectively suggests a gastroprotective effect.

The findings of Rozza et al. (37) were obviously important, as they provided evidence of a reduction in key pro-inflammatory factors and an increase in anti-inflammatory markers following menthol administration to a pathological inflammatory lesion. Similarly, Bastaki et al. (43) found similar findings when exploring the role of menthol in reducing inflammation associated with acetic acid-induced colitis in the colonic mucosa of rats. The authors utilized acetic acid as a means of inducing an inflammatory bowel disease phenotype in rats, based on specific inflammation in the colon. Acetic acid administration led to an increase in pro-inflammatory cytokines as well as marked antioxidant activity, ulceration (micro- and macroscopic), and loss of body weight in affected animals, broadly consistent with the inflammatory bowel disease phenotype seen in humans (47). Based on the results from Peiris et al. (47) and Bastaki et al. (43), menthol-related TRPM8 may be a potential anti-inflammatory mediator in irritable bowel syndrome patients for the increased TRPM8-IR on dendritic cells within the colonic mucosa coupled with decreased release of cytokines, which delineate the potential cellular mechanisms underlying the therapeutic benefit of menthol. Peiris et al. (47) thought that the increased production of mRNA and TRPM8-IR in irritable bowel syndrome was an example of an inducible anti-inflammatory mechanism that can be controlled by menthol. Following the application of menthol, it was observed that body weight reduction was attenuated, ulcer appearance improved on histopathological examination, and both antioxidant and anti-inflammatory effects were observed. Specifically, reductions in pro-inflammatory cytokines IL-1, IL-23, and TNF-α were observed. However, IL-6 showed no significant change in levels of expression or activity following menthol administration, in contrast to the study by Rozza et al. (37). The authors also evaluated levels of calprotectin, a protein that is released by pro-inflammatory leucocytes and is associated with the progression of inflammatory bowel disease (49). Calprotectin levels were reduced following menthol treatment, providing further evidence for a decrease in pathological inflammation. Merat et al. (50) observed in a randomized, double-blind, placebo-controlled clinical study that an enteric-coated peppermint-oil formulation called Colpermin reduces stomach pain (8-week therapy) and discomfort (1-week therapy). Similarly, in a randomized, placebo-controlled clinical trial, a 4-week course of Colpermin relieved irritable bowel syndrome patients’ overall abdominal symptoms (51). Harrington et al. (52) figured out how the compounds containing peppermint are reported to reduce symptoms of bowel hypersensitivity. They found that the antinociception caused by TRPM8 at peripheral sensory endings was caused by the activation of TRPM8 itself and by the effect of TRPM8 activation on the function of other TRP channels like TRPV1 and TRPA1. Both TRPM8 and TRPV1 inhibit the mechanosensory function mediated by TRPA1, and TRPM8 also interacts with TRPV1 (52). In the context of complicated TRP channel interactions, menthol, as a natural agonist of TRPM8, may act similarly to icilin, also playing a pro-and anti-nociceptive role in irritable bowel syndrome.

Both Rozza et al. (37) and Bastaki et al. (43) studies acknowledged that antioxidant activity and anti-inflammatory activity are typically related to the pathology of the gastrointestinal tract, with overlapping and linked features. This adds a complication to the observed effects of menthol, as the antioxidant activity may have driven much of the improvements seen in terms of the histopathology of lesions or the clinical status of affected animals. While this does not preclude the specific effects of menthol on the inflammatory response, it may highlight an adjunctive or indirect mechanism through which this anti-inflammatory activity occurs, *via* the reduction of reactive oxygen species or antioxidant activity (53). Separation of these pathways and further elucidation of menthol’s effects on inflammatory pathways alone may be valuable in future research. Furthermore, studies in animals with chemically-induced lesions and in *in vitro* models may not have a clear application to human conditions associated with inflammation. Indeed, conditions such as inflammatory bowel disease and ulceration are often chronic in nature and may involve a complex immune component, particularly in inflammatory bowel disease, not replicated in the chemical induction of lesions in the acute setting (47). Whether changes in the immunological (and possible inflammatory) milieu occur, which may influence the effects of menthol on inflammatory activity, remains to be seen in humans.

### Cutaneous and submucosal tissue inflammation

The application of menthol to cutaneous tissue and submucosal tissue is common in clinical practice for the alleviation of pain and to promote a cooling effect (54). However, there is increasing interest in the potential for menthol to exert anti-inflammatory effects when applied to the skin and associated tissues, including inflammation associated with pathological conditions and processes. Andersen et al. (39) reported that the application of a 40% menthol-based cream to an area of irritation and inflammation on the forearm leads to a reduction in pain, neurogenic inflammation, and hyperalgesia (high responsiveness to sensations). This study utilized topical 10% trans-cinnamaldehyde application to elicit the cutaneous symptoms, with the treatment of menthol simultaneously noted to reduce pain intensity (P<0.01), neurogenic inflammation (P<0.01), and hyperalgesia (P<0.05) compared with symptoms when menthol was not applied.

The counter-irritant effect of menthol in this instance suggests that TRPM8 agonism is a potent mechanism for overcoming pathological stimuli on skin linked to neurogenic inflammation. While the study did not evaluate specific markers of inflammation, including established cytokines or other elements of the immune response, the link between inflammation and the symptoms observed was considered supportive of the anti-inflammatory effects of topical menthol (39). The targeting of TRPM8 and associated channels in reducing chronic pain linked to inflammation and neurogenic pain suggest this is a valid approach and that menthol is a putative target compound for future investigation (55). Interestingly, it has been observed that the G-protein subunit Gα_q_ may play a role in diminishing the activity of menthol-mediated TRPM8 activation (56). The association of Gα_q_ with the TRPM8 receptor leads to conformational changes that block the activity of menthol at the receptor, reducing the potential for anti-inflammatory signaling (56, 57). This is an important finding, as it has been observed that when inflammation is established in cutaneous tissue and includes the Gα_q_-TRPM8 association, this leads to a lack of efficacy of menthol activity in reducing the effects of inflammation (57). Therefore, while simultaneous use of menthol and 10% trans-cinnamaldehyde allowed the effects of menthol to reduce inflammation and symptoms, sequential menthol application (i.e., as a treatment) may potentially lack efficacy due to this mechanism (58). More work needs to be done to clarify the factors that block menthol-TRPM8 signaling in the context of inflammation in order to establish the value of menthol as a topical therapeutic following the established onset of inflammation.

Some perspectives propose that menthol may exert its anti-inflammatory effect by interacting with the dense network of nerves that are embedded in the dermal-epidermal junction of the skin. In connection with the nerve and inflammation impact, nerve endings detect cutaneous stimulation, trigger inflammatory responses, produce vasoactive neuropeptides, and transmit pain signals (59). After an injury, neuropeptides and noradrenaline released from nerve terminals in the skin activate receptors on the surface of keratinocytes, resulting in the production of inflammatory cytokines and nerve growth factor, as well as the degranulation of mast cells. Superimposed upon that, mast cell degranulation produces several inflammatory mediators, including tryptase, proteases, histamine, cytokines, and eicosanoids, all of which are theoretically capable of producing inflammation and sensitization (60).

Despite the uncertainty over the value of topical menthol in reducing inflammation that is established in the skin, the anti-inflammatory effects of menthol have been proposed to influence wound healing, as investigated by Rozza et al. (61). Skin wound healing is a complex process that includes an inflammatory phase, whereby reductions in inflammatory markers, including IL-6, have been linked to accelerated skin wound healing (61). In this study, the authors evaluated the effects of different periods of time of treatment with menthol (3, 7, or 14 days) in rats with skin wounds, comparing collagenase-based and menthol-based creams. The menthol-based cream led to accelerated healing within the first three days of treatment, which is consistent with a reduction in the initial inflammatory phase of wound healing when compared with collagenase-based creams. Furthermore, this healing was linked to a reduction in pro-inflammatory cytokines TNF-α and IL-6 (reduced expression of mRNA). Over time, menthol-based treatment was found to not only decrease pro-inflammatory cytokine levels through the inflammatory, proliferative, and remodeling phases of wound healing.

This study is important as it demonstrates the effectiveness of the anti-inflammatory effects of menthol within the context of wound healing. However, it is notable that menthol also had effects on antioxidant activity and cellular proliferation during the healing process, suggesting that anti-inflammatory actions could not be specifically linked to improved wound healing. Furthermore, the healing of wounds in rats may not be directly applicable to human wound healing in practice, suggesting further investigations are needed to ensure the clinical relevance of this finding. It is also noteworthy that the effects of menthol were sustained over at least 14 days with no ill effects on the rats, suggesting that chronic inflammation linked to wound healing may be targeted by menthol safely (61). Further evidence of the effects of menthol on chronic inflammatory conditions is presented in the following sections, with a focus on the urinary tract, neural, and respiratory applications of the compound.

### Conditions associated with chronic inflammatory responses

Within the context of chronic inflammation, there has been an interest in the potential therapeutic properties of menthol to alleviate inflammation and antioxidant damage. A study by Shahid et al. (62) explored the role of volatile organic compounds within urine samples taken from patients with or without interstitial cystitis. The condition of interstitial cystitis is characterized by an inflammatory response affecting the bladder, leading to pronounced urinary symptoms, which are often chronic in nature (63). Examination of volatile organic compounds and comparison between those with or without interstitial cystitis identified that levels of menthol differed between the groups, with lower levels in individuals with the condition (62). This may be interpreted as lower levels of menthol correlating with higher levels of inflammatory reactions in the urinary tract. However, the establishment of causation is challenging due to the design of the study, and therefore, menthol responses may have been influenced by inflammation of other aspects of the biochemical response to cystitis. These findings are intriguing, as they suggest that menthol may be a potential anti-inflammatory agent in the urinary tract. It has been previously noted in pathological specimens of bladders with inflammatory conditions that levels of TRPM8 expression are elevated in such situations compared to healthy bladders (64). Therefore, the increase in these receptors may play a role in sensitization and symptoms, while potentially offering an opportunity for targeting with menthol. Hence, the data suggest that menthol exposure in the urinary tract may be associated with anti-inflammatory effects, potentially mediated by TRPM8.

Similar to chronic inflammation seen in other tissues, neuroinflammatory responses are linked to a range of disease states, including dementia (65). The protective effects of menthol administration have also been observed in the context of Parkinson’s disease models (44). Specifically, menthol administration has been shown to be protective against lipopolysaccharide-induced inflammatory damage to dopaminergic neurons, which are characteristically affected by Parkinson’s disease pathology (44). Menthol demonstrated an anti-inflammatory effect, with inhibition of pro-inflammatory enzymes and cytokines *via* activation of the nuclear factor kappa B (NF-κB) and p38 mitogen-activated protein kinase (MAPK) pathways. During the neuroinflammatory process, Du et al. (44) found that menthol inhibited the increase in phosphorylation levels of p65, ERK1/2, JNK1/2, and AKT in an *in vitro* experiment. Menthol has been shown to increase the activation of dopaminergic neurons, but also decrease neuronal firing in cellular studies, suggesting that additional effects on the stimulation of dopaminergic neurons may also be relevant in Parkinson’s disease (66, 67). In terms of Alzheimer’s disease, research indicates that menthol therapy that pharmacologically raises body temperature also results in a reduction in tau phosphorylation in the brain, but not in conjunction with an inflammatory response (68). However, this is an area that requires further exploration, given the relative paucity of literature on menthol’s effects on neuroinflammatory pathways.

### Lung inflammation

Despite the published anti-inflammatory effects of menthol within experimental settings, there is evidence to suggest that in specific contexts, menthol may have pro-inflammatory effects or may induce inflammatory responses. This has been noted in the context of cigarette smoking, when inflammatory activity in human lung epithelial cells has been assessed (32). Specifically, Lin et al. (32) found that menthol cigarette smoke exposure led to more severe lung inflammation than non-menthol smoke inhalation in the context of sub-chronic exposure. With exposure for up to seven days, it was noted that lung inflammation was more severe when menthol smoke was inhaled, based on activation of epithelial and lung MAPK pathways *via* TRPM8, along with histopathological markers of lung inflammation. The authors also found that treatment with a TRPM8 inhibitor suppressed MAPK activation and subsequent lung inflammation (32). These findings build on previous *in vitro* work that suggested that exposure to menthol cigarette smoke increased reactive oxygen species sensitivity in lung parenchymal cells, increased TRPM8-mediated MAPK pathway activation, and promoted inflammatory responses to a greater degree than non-menthol smoke exposure.

Sundar et al. (69) and Kaur et al. (70) have noted that tobacco flavoring can influence the inflammatory response of cigarette smoke inhalation in the lungs. A variety of flavors added to tobacco have been associated with decreased viability of cells, decreased cell numbers in cultures, and increased levels of inflammation after exposure compared with unflavored tobacco, including when flavored with menthol (69). It is proposed that menthol acts on the TRPA1 receptor to activate an inflammatory response in lung parenchyma based on cell experiments and animal models of cigarette smoke inhalation (30, 71). Markers of inflammation elevated with menthol flavored smoke inhalation included cyclo-oxygenase-2 (COX-2) and prostaglandin levels, which are recognized as drivers of an acute local inflammatory reaction in various tissue types (30).

The findings of Lin et al. (32) contrast with those observed by Liu et al. (42), who investigated the effects of menthol (L-menthol) on cigarette smoke extract (CSE) induced lung injury in rats. The study found that following CSE injury, which is characterized by acute oxidative and inflammatory damage to the lung tissue, administration of menthol led to a marked reduction in the inflammatory response. Specifically, IL-1β, IL-6, and TNF-α were downregulated following the activation of the NF-кB and MAPK pathways (42). Liu et al. (72) had previously observed that menthol administration suppressed hypersensitivity to cigarette smoke in the context of a chronic inflammatory state, validating these observations.

The contrasting findings of menthol exposure on inflammatory responses in smokers may be dependent on a range of effects. For instance, it has been noted that menthol may alter the metabolism of nicotine, increasing systemic exposure to nicotine and slowing the metabolic clearance of nicotine and associated compounds in the lungs (35). Whether this increases the potential for more inflammation, which is associated with lung nicotine exposure, remains to be confirmed, but is one hypothesis as to why co-administration of menthol and nicotine may lead to heightened inflammation (70). Besides, the difference in constituents between commercial mentholated and non-mentholated cigarettes may also introduce confounding factors in the experiment.

Furthermore, it has been suggested that chronic smokers may have altered sensory irritation responses as a consequence of pulmonary remodeling and inflammatory reactions that are long-standing in the respiratory tract (70, 73, 74). The sensitivity of TRPA1 and TRPV1 receptors has been shown to be modified in chronic smokers, which may diminish the potential for menthol to act on local receptors to reduce inflammation (75, 76). Additionally, a chronic state of inflammation within the respiratory tract may induce changes to receptor sensitivity and responses to stimulation, reducing the anti-inflammatory effects of menthol (74). Indeed, in the research by Lin et al. (32), sub-chronic smoke exposure in mice (less than seven days) was sufficient to promote additional inflammatory reactions with menthol exposure, while this was not observed in acute exposure (20 minutes). This suggests that shorter periods of exposure may not be linked to additive inflammatory outcomes with menthol exposure (32). Therefore, the chronicity of smoking and the complex biochemical pathways that occur following exposure to menthol and cigarette smoke extracts (including nicotine) may account for variations in inflammatory responses to menthol in published research.

It should also be noted that experimental models using animals exposed to cigarette smoke may differ from exposure in humans. Mice are obligate nasal breathers, and hence cigarette smoke extracts and/or inhaled menthol interact with nasal receptors predominantly (77). In contrast, human smokers inhale cigarette smoke compounds through the mouth, allowing exposure predominantly in the lungs and oral cavity (30). Therefore, differential responses in inflammation and other symptoms (cough or irritation) may be accounted for by differences in anatomy, sequential receptor activation, and other mechanisms (70).

## Effect of TRP channels on the anti-inflammatory effect of menthol

### Introduction to the TRP Family

TRP channels were first identified in a mutant breed of the fruit fly Drosophila, known as “transient receptor potential” in 1969 (78). From the control of cell motility and phagocytosis to the generation and release of inflammatory mediators, TRP channel-mediated effects on immune cells are undoubtedly numerous. Menthol has the potential to become a novel treatment for inflammatory illnesses due to the wide functional significance of TRP channels in inflammation and immunity (79). This section provides information on the role of TRP channels associated with menthol, such as TRPM8, in inflammatory diseases.

### TRPM8 (Transient receptor potential cation channel subfamily melastatin member 8)

TRPM8, also known as the cold and menthol receptor 1 (CMR1), is a protein encoded by the TRPM8 gene in humans. It is generally known that menthol is the agonist of TRPM8. The well-known chilling feeling that menthol causes when breathed, consumed, or applied to the skin is brought about by its capacity to chemically activate the cold-sensitive TRPM8 receptors in the skin. However, TRPM8 appears to perform far more functions than simply bringing coldness (6, 55, 79). As mentioned above, menthol-induced TRPM8 also plays an important physiological role in inflammation.

Some researchers used various TRPM8 antagonists, such as AMTB and AMG2850, indicating that menthol-induced TRPM8 has inflammation-related physiological functions. Liu et al. (72) found that the suppressive effect of menthol on laryngeal C-fiber hypersensitivity to cigarette smoke could be reversed by pre-treatment with AMTB, suggesting that it is mediated through TRPM8. Ha et al. (30) observed that the counter-irritant effect of L-menthol against cigarette smoke was blocked by the TRPM8 antagonist AMG2850, supporting a role for TRPM8 receptor pathways in this effect. Moreover, the effect of L-menthol, which can strongly attenuate the sensory irritation response to acrolein, was significantly diminished by the TRPM8 antagonist (71). When it comes to the TRPM8^-/-^ animal models, Ramachandran et al. (80) used knockout mice, showing that activation of TRPM8 with a kind of agonist named icilin attenuates the inflammatory response.

Studies showed that TRPM8 might affect in inflammation process by mediating the release of inflammatory cytokines (47, 81). NF-κB mediated TRPM8’s TNF-α inhibition (81). The study found that TRPM8 expression enhanced TRPM8-NF-κB interaction, decreasing NF-κB nuclear localization. On this basis, the suppression inhibited TNF-α gene transcription. TRPM8 agonists, such as menthol and icilin, are found to decrease the release of cytokines IL-1β, IL-6, and TNF-α from dendritic cells (47).

As effectual as the TRPM8’s anti-inflammatory effect may seem, however, there are still some doubts about it at present. In an *in vitro* study, human bronchial epithelial cells’ activation of TRPM8 promotes the expression of IL-6, IL-8, and TNF-α (82). TRPM8 mRNA and protein expression in bronchial tissues were upregulated by cigarette smoke extract. Based on the upregulation, cigarette smoke extract synergistically amplifies inflammatory factor release (82). Furthermore, many researchers hold a critical opinion on TRPM8’s anti-inflammatory effect, indicating that the relationship between TRPM8 and inflammation remains to be further discussed (70). Seemingly, not only does TRPM8 have an anti-inflammatory effect, but it also plays a role in pro-inflammatory properties under some circumstances.

### Other channels of the TRP subfamily related to menthol

Except for TRPM8, there are other channels of the TRP subfamily, including TRPA1, TRPV1, and TRPV3, which can also be influenced directly or indirectly by menthol, and are seemingly involved in the process of menthol’s anti-inflammatory effect.

As a member of the TRP subfamily of channels, the TRPA1 is largely concentrated in a subpopulation of primary sensory neurons of the trigeminal, vagal, and dorsal root ganglia, where its activation promotes neurogenic inflammatory responses (83). TRPA1 detects pro-inflammatory molecules. Meanwhile, it can also be activated by endogenous pro-inflammatory molecules in several inflammatory diseases, including asthma, rheumatoid arthritis, and dermatitis (83, 84). In terms of the activation of TRPA1 in murine by menthol, submicromolar to low-micromolar menthol doses activate heterologously produced TRPA1 channels, while greater amounts block them (85–87). Andersen et al. (40) hold the viewpoint that high-concentration menthol can reduce the neurogenic inflammation caused by TRPA1 agonist trans-cinnamaldehyde. Harrington et al. (52) reveal that icilin inhibits the mechanical hypersensitivity generated by AITC-induced TRPA1 activation. The absence of icilin’s impact on mechanical desensitization was also seen in mice lacking the TRPA1 gene. During the past few decades, much work has been done to investigate the connection between TRPM8 and TRPA1 in terms of the anti-inflammatory effect (15). TRPM8 mediates inflammation through crosstalk with TRPA1. In addition, through the activation, TRPM8 reduces the activity of TRPA1 channels and thus inhibits mechanosensitivity (52). But some research seems to disregard the bimodal action of menthol on murine TRPA1 channels. There might be a complex anti-inflammation effect regulated by both TRPM8 and TRPA1, because of menthol’s activation of TRPM8 and inhibition of TRPA1 (87). It’s not hard to see that, similar to TRPM8, TRPA1 also plays a complex role in inflammation. Thus, more comprehensive studies are needed to understand menthol’s effect on TRPA1 as a potential anti-inflammatory and analgesic therapy.

TRPV1, which is activated by a variety of endogenous and exogenous substances, appears to be the most well-researched TRPV channel. As far as we know, TRPV1 has been linked to both processes that cause inflammation and those that stop it. This suggests that it is important in inflammation. Depending on the various inflammatory sites and conditions, neuronal TRPV1 expression and function are drastically changed. TRPV1 not only plays a pro-inflammatory role in diseases such as pancreatitis, dermatitis, and arthritis (88–91). But interestingly, TRPV1 also has an anti-inflammatory effect in conditions including allergic airway inflammation and acute lung injury (92). Speaking of the interaction of menthol and TRPV1, it seems that menthol can’t activate or inactivate TRPV1 directly (87). Growing evidence shows that TRPM8 might reduce the release of calcitonin gene-related peptide and inflammatory factors activated by TRPV1, indicating that TRPM8 may perform an anti-inflammatory role to counteract TRPV1’s pro-inflammatory effects (29, 80).

TRPV3 functions in a variety of processes, including temperature sensation and vasoregulation. It has wide tissue expression that is especially high in the skin (keratinocytes) but also in the brain. It functions as a molecular sensor for innocuous warm temperatures. Mice lacking the protein are unable to sense elevated temperatures (>33°C) but are able to sense cold and noxious heat (93). In addition to thermosensation, TRPV3 channels seem to play a role in hair growth because mutations in the TRPV3 gene cause hair loss in mice. Based on the current evidence, TRPV3 may be involved in skin inflammation (94). Through the research of Szöllősi et al. (95), TRPV3 is found to be functionally expressed in human epidermal keratinocytes and is involved in cutaneous inflammatory processes. Szántó et al. (96) regarded TRPV3 as an undocumented negative regulator of sebaceous lipid synthesis with a significant pro-inflammatory impact. Data on the effect of menthol on TRPV3 channels is rather limited. There are also a few studies showing that TRPV3 can be attenuated to induce the inflammation of other tissues, such as the cardiovascular system (97). The differences between applying menthol and applying TRPV3’s agonist to skin inflammation remind us of an unclear overlapping effect. Hence, further clinical studies are needed to investigate the overlap of the receptor pharmacology between these channels after the application of menthol to certain inflammatory skin conditions.

## Conclusion

In conclusion, it is seemingly that menthol administration in a great variety of experimental models and settings is associated with anti-inflammatory activity. This activity is characterized by a decrease in pro-inflammatory cytokines and related inflammatory markers, including those potentially linked to chronic inflammation, as well as pathway activation that is linked to the regulation of inflammatory enzymes and proteins. Furthermore, menthol administration in pathological inflammatory contexts appears to have favorable outcomes on histopathological characteristics of lesions and wider biological effects. As menthol is considered a safe agent for humans, given the widespread use and medical indications for menthol-containing products at present, the therapeutic potential of menthol as an anti-inflammatory can justifiably be considered on the basis of this review.

Taken together, studies exploring the protective effects of menthol against inflammatory damage, or association with protection against chronic inflammation, are indicative of potential therapeutic applications of the compound. However, it is vital to note the limitations of the studies completed to date. One main limitation is that most of the studies are either *in vitro* or based on animal models of human disease, which may limit our direct application to human pathology. While menthol has been extensively used therapeutically in humans, demonstration of a clear anti-inflammatory effect should be sought and evaluated using biochemical markers or inflammation in a pathological context. Furthermore, while the evidence for the anti-inflammatory effects of menthol is compelling, additional insights may be needed to clarify the mechanisms of action and the clinical relevance of any anti-inflammatory effects. This is especially noteworthy considering the evidence for the pro-inflammatory impact of inhaled menthol when combined with cigarette smoking (42). Inflammatory cascades are complex and closely related to immunological function and antioxidant activity in multiple tissues; further delineation of these mechanisms and pathways is needed to truly appreciate the potential for menthol, which appears to be a molecule with multiple cellular targets, as a therapeutic anti-inflammatory compound.

## Author contributions

HC: Conceptualization, Data Curation, Project Administration, Writing - Original Draft. XA: Conceptualization, Funding Acquisition, Writing - Review & Editing. All authors contributed to the article and approved the submitted version.

## Funding

The author would like to thank the support of National University Student Innovation Program of China from Chengdu University of Traditional Chinese Medicine (No. 202110633026).

## Conflict of interest

The authors declare that the research was conducted in the absence of any commercial or financial relationships that could be construed as a potential conflict of interest.

## Publisher’s note

All claims expressed in this article are solely those of the authors and do not necessarily represent those of their affiliated organizations, or those of the publisher, the editors and the reviewers. Any product that may be evaluated in this article, or claim that may be made by its manufacturer, is not guaranteed or endorsed by the publisher.
